# Bilateral Diffuse Anterior Scleritis With Ocular Hypertension Revealing Rheumatoid Arthritis

**DOI:** 10.7759/cureus.114247

**Published:** 2026-08-10

**Authors:** Moulahid Loubna, Fatimazahra Liqali, Soukaina Tidrarine, Nabil Bouslous, Moustaine M Omar

**Affiliations:** 1 Ophthalmology, Mohammed VI University Hospital, Agadir, MAR; 2 Rheumatology, Mohammed VI University Hospital, Agadir, MAR; 3 Faculty of Medicine and Pharmacy, Ibn Zohr University, Agadir, MAR; 4 Ophthalmology, University Hospital Souss-Massa, Agadir, MAR

**Keywords:** anterior diffuse scleritis, intraocular pressure, ocular hypertension, ocular inflammation, rheumatoid arthritis

## Abstract

Rheumatoid arthritis is a chronic systemic autoimmune disease that may involve extra-articular organs, including the eye. Scleritis is a severe and potentially sight-threatening ocular manifestation frequently associated with rheumatoid arthritis. We report the case of a 63-year-old male presenting with a 1-month history of bilateral red and painful eyes associated with photophobia and headache, initially misdiagnosed as glaucoma due to elevated intraocular pressure. Ophthalmologic examination revealed decreased visual acuity, diffuse conjunctival hyperemia, and intraocular pressure of 30 mmHg in both eyes. Laboratory investigations confirmed seropositive rheumatoid arthritis. The patient was treated with systemic and topical corticosteroids along with acetazolamide, leading to significant clinical improvement and reduction in intraocular pressure. This case highlights the importance of considering inflammatory etiologies in patients presenting with ocular hypertension and emphasizes the need for early diagnosis and multidisciplinary management to prevent vision-threatening complications.

## Introduction

Rheumatoid arthritis (RA) is a chronic systemic autoimmune disease that primarily affects the joints but may also involve extra-articular organs, including the eye [1]. Ocular manifestations are diverse, ranging from relatively benign conditions, such as keratoconjunctivitis sicca and episcleritis, to severe, vision-threatening diseases such as scleritis, peripheral ulcerative keratitis, and uveitis [2].

Scleritis is a serious inflammatory condition of the sclera, most commonly associated with systemic autoimmune diseases, particularly rheumatoid arthritis [1,2]. Anterior diffuse scleritis is the most frequent form and is characterized by severe ocular pain and redness, with a risk of complications if not promptly treated. In some cases, scleritis may be associated with elevated intraocular pressure, which may mimic glaucoma and potentially delay appropriate management.

We report a case of bilateral anterior diffuse scleritis with elevated intraocular pressure revealing underlying rheumatoid arthritis.

## Case presentation

Patient information and timeline

A 63-year-old male presented with a 1-month history of bilateral red and painful eyes associated with photophobia and headache. The pain radiated to the temples, awakened him at night, and responded poorly to analgesics. His past medical history was notable for pulmonary tuberculosis treated 25 years earlier and inflammatory arthritis managed with deflazacort for one year. He had no prior ocular history.

Initially, the patient was treated for glaucoma due to elevated intraocular pressure; however, symptoms persisted, prompting referral to our department.

Clinical findings

**Figure 1:**
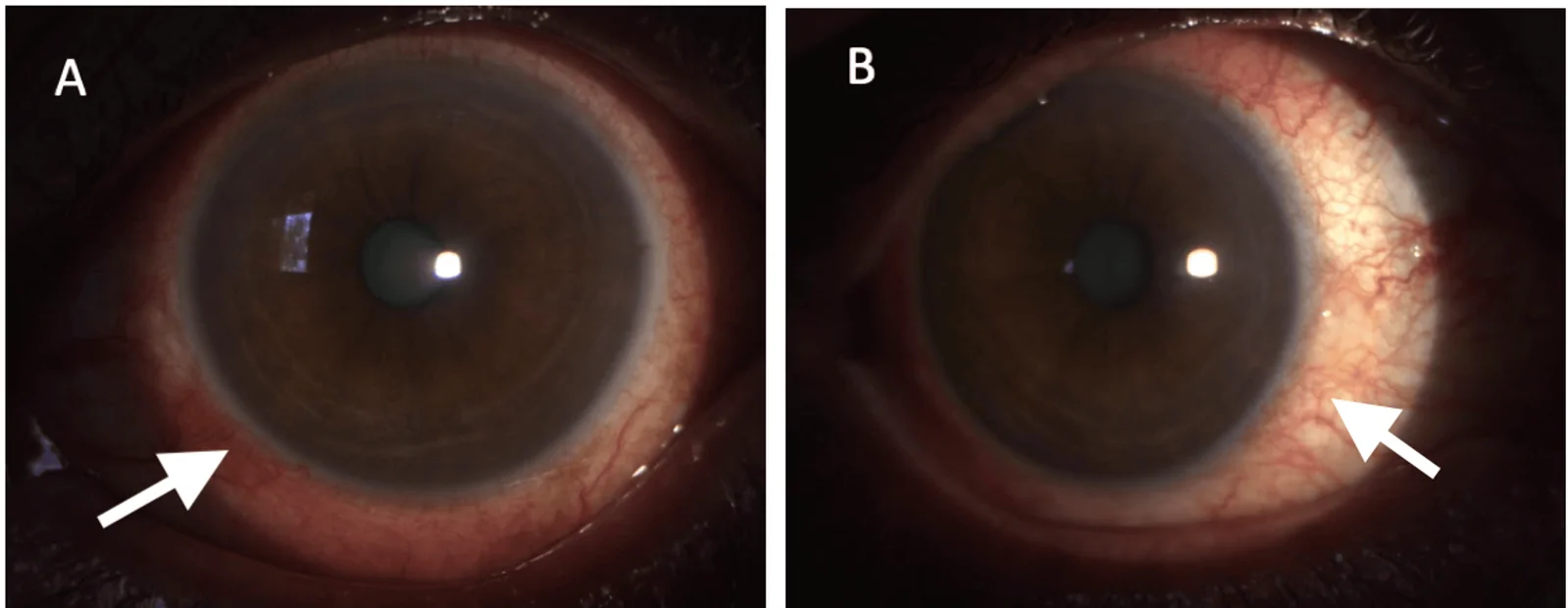
Bilateral diffuse conjunctival hyperemia (A) right eye; (B) left eye; arrows indicate scleral involvement

Best-corrected visual acuity was 0.40 logMAR in both eyes. Ophthalmologic examination revealed deep and diffuse conjunctival hyperemia with scleral and episcleral injection in both eyes (Figure 1). The corneas were clear, with no keratic precipitates, and the anterior chambers were quiet, with no posterior synechiae in either eye. A bilateral nuclear cataract grade 3 was noted.

Intraocular pressure, measured by Goldmann applanation tonometry, was 30 mmHg in both eyes. Gonioscopy revealed open anterior chamber angles bilaterally, with no peripheral anterior synechiae. Fundus examination was unremarkable. Instillation of 10% topical phenylephrine did not result in blanching of the deep episcleral vessels, supporting the diagnosis of scleritis.

Diagnostic assessment

Bilateral B-scan ultrasonography was unremarkable and showed no evidence of posterior scleritis or posterior segment involvement. Laboratory investigations showed evidence of systemic inflammation and autoimmune activity, including elevated C-reactive protein and positive anti-cyclic citrullinated peptide antibodies (Table 1). Serologic testing for HIV, hepatitis B, and hepatitis C was negative. Further rheumatologic evaluation confirmed destructive seropositive rheumatoid arthritis. Based on the clinical and laboratory findings, a diagnosis of bilateral anterior diffuse scleritis associated with ocular hypertension secondary to rheumatoid arthritis was established.

**Table 1:** Laboratory findings WBC: white blood cells, Hb: hemoglobin, CRP: C-reactive protein, anti-CCP: anti-cyclic citrullinated peptide antibodies, AST: aspartate aminotransferase, ALT: alanine aminotransferase, HIV: human immunodeficiency virus, HBsAg: hepatitis B surface antigen, anti-HCV serology: antibody to hepatitis C virus

Test	Result	Reference range	Units
WBC	10 650/mm3	4000-11000	/mm3
Hb	14.7	13-18	g/dL
Platelets	263 000	150000-400000	/mm3
C-reactive protein (CRP)	18.8	< 6	mg/L
Erythrocyte sedimentation rate (ESR)	40	<20	mm/h
Anti-cyclic citrullinated peptide (anti-CCP) antibodies	186.8	< 20	U/mL
Rheumatoid factor latex test	114	<12	IU/mL
WAALER-ROSE test	57	<6	IU/mL
Creatinine	0.71	0.4-1.3	mg/dL
AST	12	0-40	U/L
ALT	9	0-45	U/L
HIV serology	0.11	<1	S/Co
HBsAg	0.10	<1	-
Anti-HCV serology	0.05	<1	-

Therapeutic intervention

The patient received systemic corticosteroids (deflazacort 30 mg/day) as bridging therapy prior to the initiation of immunosuppressive treatment by the rheumatology department. Topical dexamethasone 0.1% (Dexafree®) was prescribed to control ocular inflammation. Intraocular pressure elevation was managed with oral acetazolamide 250 mg twice daily for 5 days.

Follow-up and outcomes

The patient showed significant clinical improvement, with resolution of pain, redness, and photophobia. Intraocular pressure decreased from 30 mmHg to 17 mmHg. At the one-month follow-up, best-corrected visual acuity improved to 0.15 logMAR in both eyes, with residual visual limitation related to bilateral nuclear cataract. Intraocular pressure remained controlled with topical dorzolamide monotherapy after discontinuation of oral acetazolamide. Topical dexamethasone was gradually tapered following control of ocular inflammation. No recurrence of scleritis was observed during the one-month follow-up period.

## Discussion

Rheumatoid arthritis is a chronic systemic autoimmune disease that primarily affects peripheral joints but frequently involves extra-articular organs, including the eye [1]. Ocular manifestations range from benign conditions, such as keratoconjunctivitis sicca and episcleritis, to severe, vision-threatening diseases such as scleritis, peripheral ulcerative keratitis, anterior uveitis, and retinal vasculitis [2].

Scleritis is a severe inflammatory condition of the sclera, most commonly associated with systemic autoimmune diseases, particularly rheumatoid arthritis [2]. In a retrospective study of 500 patients with scleritis, rheumatoid arthritis was identified as the most frequent associated systemic disease among connective tissue and vasculitic disorders [3], highlighting the importance of a thorough systemic evaluation.

Scleritis is characterized by scleral edema and inflammatory cell infiltration and is classified into anterior and posterior forms. Anterior scleritis is further divided into necrotizing and non-necrotizing types, with the latter including diffuse and nodular subtypes [4].

Anterior diffuse scleritis, as observed in our patient, is the most common form and is characterized by severe ocular pain, redness, and photophobia, often worsening at night. If untreated, inflammation may extend to adjacent ocular structures, leading to complications such as uveitis, cataract, elevated intraocular pressure, peripheral ulcerative keratitis, and, in severe cases, scleral thinning or globe rupture [5].

Elevated intraocular pressure is a recognized complication of scleritis and may result from inflammatory damage to the trabecular meshwork, impaired aqueous humor outflow, or increased episcleral venous pressure. A retrospective study reported intraocular hypertension in 12.6% of eyes with scleritis, particularly in anterior forms [6], while higher rates have been reported in other series [7]. Ocular hypertension can occur in all types of scleritis but appears more frequent in necrotizing disease [8].

In the present case, elevated intraocular pressure initially led to a misdiagnosis of glaucoma, delaying appropriate management. This highlights the importance of considering inflammatory etiologies in patients presenting with ocular hypertension associated with ocular pain and redness.

Management of diffuse anterior scleritis relies on systemic anti-inflammatory therapy as first-line treatment [4]. Topical medications alone are generally insufficient [9]. Systemic corticosteroids are typically used at low doses for a limited duration due to their potential side effects. A multidisciplinary approach is essential, focusing not only on controlling ocular inflammation but also on treating the underlying rheumatoid arthritis with disease-modifying antirheumatic drugs, which improve both articular and extra-articular manifestations [10].

Treatment strategies depend on disease severity. Oral nonsteroidal anti-inflammatory drugs (NSAIDs) are the first-line therapy in non-necrotizing scleritis. In cases of inadequate response or more severe presentations, systemic corticosteroids, either oral or intravenous, are indicated. In necrotizing forms or when associated with systemic disease, immunosuppressive agents are often required and have shown significant efficacy. In refractory cases, biologic therapies, particularly anti-TNF agents, are increasingly used [9,10].

## Conclusions

Anterior diffuse scleritis is a severe ocular manifestation of rheumatoid arthritis and may be associated with elevated intraocular pressure. This bilateral case shows that ocular hypertension with painful red eyes should raise suspicion of an inflammatory cause, not only glaucoma. Early diagnosis and multidisciplinary management are essential to control inflammation, treat the underlying systemic disease, and prevent vision-threatening complications.
